# Supplementary material for: Study on key parameters of buckling deformation instability and fracture of rock beams and asymmetric distribution law of stope stress
Source: PLoS One. 2024 Jun 11;19(6):e0305244. doi: 10.1371/journal.pone.0305244 (PMC11166296; doi:10.1371/journal.pone.0305244)
Supplement: Attachment — Submitted filename: Response to Reviewers.docx [file pone.0305244.s002.docx]

Thank you for your letter and for the reviewer’s comments concerning our manuscript entitled “Study on the key parameters of buckling deformation instability and fracture of rock beams and asymmetric distribution law of stope stress” (ID: PONE-D-23-41833). Those comments are all valuable and very helpful for revising and improving our paper, as well as the important guiding significance to our research. We have studied comments carefully and have made corrections which we hope to meet with approval. Revised portions are highlighted in yellow on the paper. The main corrections in the paper and the responses to the reviewer’s comments are as following:

**Reviewer 1:**

1. Please simplify the title of Table 1. It is proposed that the title of Table 2 be changed to Similar material ratio of Shaqu Mine. Please make the same changes to Table 3 and Table 4.

Response 1: We think this is an excellent suggestion. According to the reviewer's comments, we have revised the title of Tables 1, 2, 3, and 4. The revised contents are on lines 145, 152, 153, and 154 of the revised manuscript.

2. It is suggested that the title of section 2.2 be changed to simulate experimental conditions more in line with the topic. The reason for the selection of the experimental model similarity ratio is not sufficient, not only based on the size of the experimental bench.

Response 2: We agree with the reviewer’s assessment. According to the reviewer's comments, we have made changes in the manuscript. The basis of similarity ratio selection is explained. The revised contents are on lines 134-142 of the revised manuscript.

3. Please adjust the font of the caving angle of the sketch in Figure 2d for a clearer and more aesthetically pleasing presentation.

Response 3: Thanks for your suggestion. According to the reviewer's comments, we have changed the font in Figure 2d to make it to convey the message more clearly.

4. The language and structure of the paper can be improved by further editing for clarity and coherence.

Response 4: Thanks for your constructive suggestion. We have carefully scrutinized the manuscript and made some corresponding revisions including grammatical errors and long sentences.

**Reviewer 2:**

1. What should be specified as the basis for determining the model similarity ratio, not just the basis for determining the geometric similarity ratio.

Response 1: Thanks for your suggestion. According to your nice suggestions, we have made changes. The basis of the model similarity ratio selection is explained. The revised contents are on lines 135-142 of the revised manuscript.

2. It is recommended to unify the font sizes of all images in Figure 2 to improve the aesthetics of the paper.

Response 2: We agree with the reviewer’s assessment. We have made a uniform modification of the font size of Figure 2 to make it more consistent with the requirements.

3. In Section 3.3, the expression of “thick rock beam” in titles 3 and 4 is not accurate, which is inconsistent with the above.

Response 3: Thanks for your suggestion. According to the reviewer's comments, we have made changes in the manuscript. The revised contents are on lines 377 and 388 of the revised manuscript.

4. It is recommended to increase the number of references to supplement the basis of argumentation.

Response 4: We feel great thanks for your professional review work on our article. We have added more references to make the argument more comprehensive. The newly added references are labeled 5, 6, 9, 15, and 23.

5. There are some language problems in the paper, which need to be polished and modified by native English speakers.

Response 5: Thanks for your suggestion. We have invited a friend who is a native English speaker from the USA to help polish our article. And we hope the revised manuscript could be acceptable to you.

Yours sincerely,

Corresponding author

Zhanshan Shi

School of Mining, Liaoning Technical University, Fuxin 123000, China

<Tel:+86> 15042556003

E-mail: shizhanshan@163.com
